# Therapeutic effect and mechanism of Yougui Wan in rats with intervertebral disk degeneration

**DOI:** 10.1186/s13018-024-04554-w

**Published:** 2024-01-25

**Authors:** She Ma, Kan Liu, Jing-yan Yang, Ren-jun Huang, Dong Yu

**Affiliations:** 1 Beijing University of Chinese Medicine, Beijing, 100029 China; 2 Department of Orthopedics, Beijing University of Chinese Medicine Third Affiliated Hospital, Beijing, China

**Keywords:** Intervertebral disk degeneration, Yougui Wan, Notch signaling pathway, Inflammatory response, Nucleus pulposus cells

## Abstract

**Objective:**

To explore the potential mechanism of Yougui Wan on deformed lumbar intervertebral disk structure in rats.

**Methods:**

Thirty male Sprague–Dawley rats were randomly divided into 3 groups, with 10 rats in each group. The animals in the blank control group were healthy rats without specific treatment, and those in the model group and traditional Chinese medicine (TCM) group were used to establish the intervertebral disk degeneration (IDD) model by puncturing the annulus. Four weeks after modeling, rats in the TCM group were administered Yougui Wan by gavage for 2 consecutive weeks. Serum interleukin-6 (IL-10), macrophage migration inhibitory factor (MIF) and tumor necrosis factor alpha (TNF-*α*) levels were measured by ELISA, and the protein expression levels of collagen II and Notch1 in intervertebral disk tissues were examined by Western blotting. Apoptosis was detected by the TUNEL method.

**Results:**

Compared with those in the blank group, IL-10, MIF and TNF-*α* levels in the model group and TCM group were increased (*P* < 0.05), the protein expression levels of collagen II were decreased, and the protein expression levels of Notch1 were increased. Compared with those in the model group, the levels of IL-10 in the TCM group were increased (*P* < 0.05), the levels of MIF and TNF-*α* were decreased (*P* < 0.05), the protein expression levels of collagen II were increased, and the protein expression levels of Notch1 were decreased.

**Conclusion:**

Yougui Wan can inhibit the inflammatory response in IDD rats, reduce the degradation of extracellular matrix, reduce apoptosis in nucleus pulposus cells, and alleviate intervertebral disk degeneration. The mechanism may be related to the regulation of the Notch signaling pathway.

## Introduction

Intervertebral disk degeneration (IDD) is the main cause of a series of clinically common spinal diseases, such as intervertebral disk herniation and degenerative scoliosis. When the intervertebral disk is destroyed [1], one of the characteristics of the disease is that nucleus pulposus cells suffer from a series of catabolic cascade events at the molecular level [2], leading to the upregulation of proinflammatory cytokines [3], an increase in degradation enzymes, and a reduction in extracellular matrix synthesis [4, 5]. This catabolic process is mediated by many cytokines [6]. According to previous clinical observations by our team, Yougui Wan can effectively relieve pain and other related clinical symptoms caused by lumbar disk herniation and degeneration. After the degeneration associated with intervertebral disk injury, Yougui Wan protects the degenerative intervertebral disk by regulating the inflammatory response of the intervertebral disc. Similarly, the related key proteins and genes of the Notch pathway are involved in the repair and protection of intervertebral disks [7, 8]. Therefore, in this study, the representative factors IL-10, TNF-*α*, MIF, the Notch pathway protein Notch1, and the extracellular matrix component collagen II, which are closely related to inflammation, were selected as the study focuses.

It is extremely important to study the treatment or mitigation of IDD [9–11]. However, limitations still exist in the treatment of IDD. In the treatment of early degeneration of the intervertebral disc, there are still no good prevention or control methods. In the early stage of IDD, conservative therapy is often used in clinical practice to improve symptoms through anti-inflammatory or analgesic drugs or physical therapy. However, these methods are not long lasting, and the use of drugs in the long term might result in adverse reactions [12]. In the late stage, surgical treatment is typically used. Although surgery can quickly relieve pain, the illness still cannot be completely cured. More importantly, the structural damage caused by surgery might cause the adjacent vertebral joints to degenerate more quickly [13]. Therefore, it is critical to study the pathogenesis of IDD and the role of drugs in the prevention and treatment of IDD.

Chinese medicine also provides a deep understanding of IDD. Yin and Yang are two aspects of the nature of things that are both interrelated and opposite. The gasification law of Yin and Yang mainly lies in “Yang transforming qi” and “Yin forming.” According to the metabolic concept of modern medicine, the process of energy metabolism in metabolism is summarized as “Yang changes qi,” Yangqi insufficiency is considered an important internal cause of tissue degeneration and deficient repair. Yougui Wan, which is a representative prescription for warming therapy, is widely used to treat IDD, and its therapeutic effect lies in not only alleviating symptoms but also promoting healthy qi, improving immunity, boosting self-repair, and preventing and controlling the disease. In addition, the effect of this treatment is so remarkable that the patient's symptoms are obviously improved, and the recurrence rate is low. However, the mechanism is still unclear. Therefore, the purpose of this study was to observe the effect of Yougui Wan on a rat IDD model to explore the mechanism of Yougui Wan in the treatment of IDD.

## Materials

### Experimental animals

Thirty healthy male SD rats (SPF level; weighing 220 ± 30 g; 6–8 weeks of age; animal production license number: SCXK (Beijing) 2021–0011) were supplied by the Medical Experimental Center of the China Academy of Chinese Medical Sciences. The animals were maintained at a temperature of 23–25 °C and humidity of 50–65%, standard rat food and water were provided, and the experiments were carried out after 1 week of adaptive feeding. All procedures involving experimental animals were approved by the University Committee on the Use and Care of Animals at Beijing University of Traditional Chinese Medicine.

### Main drugs, reagents and instruments

The composition of Yougui Wan includes Radix Rehmanniae Praeparata (24 g), Deerhorn glue (12 g), yam (12 g), Eucommia (12 g), Chinese dodder seed (12 g), dogwood fructus (9 g), *Angelica sinensis* (9 g), matrimony vine (9 g), aconite (6 g), and cinnamon (6 g). The drugs were purchased from the Department of Pharmacy of the Third Affiliated Hospital of Beijing University of Traditional Chinese Medicine. The ingredients were decocted and made into Yougui Wan liquid at a concentration of 2 g/mL and stored in a refrigerator at 4 °C.

The rat interleukin 10 ELISA kit, rat macrophage migration inhibitory factor ELISA kit, rat tumor necrosis factor *α* ELISA kit, rat macrophage colony stimulating factor ELISA kit, Notch1 antibodies (CST, cat. no. 3608), collagen II antibodies (Abcam, cat. no. Ab34712), GAPDH antibodies (Abcam, cat. no. ab181602), and goat anti-rabbit IgG + HRP (Abcam, cat. no. Ab6721) were purchased from Shanghai Jianglai Biotechnology Co., Ltd.

An automatic microplate reader (model: ELx800) was purchased from BioTek, USA, and a carbon dioxide incubator (model: 3111) was purchased from Thermo, USA. The double vertical electrophoresis tank (model: MP-8001), transfer tank (model: MP-3030), and electrophoresis apparatus (model: PP-1150) were purchased from Beijing Kaiyuan Xinrui Instrument Co., Ltd. The decolorization shaker (model: TS-2000A) was purchased from Haimen Kylinbell Instrument Manufacturing Co., Ltd.

## Methods

### Preparation of the rat model

This study used the annulus puncture method to establish an IDD model [14]. The rats were adaptively fed for 1 week. Before the operation, the rats were fasted for 12 h, weighed, and anesthetized with an intraperitoneal injection of 1.5 mL/kg 3% pentobarbital sodium. After the onset of anesthesia, the hair in the spinal area of the target segment was shaved. After being cleaned and disinfected, the rats were fixed in the supine position. A 0.5-cm longitudinal incision was made on the right side of the anterior median line to expose the posterior abdominal wall and protect the intestine and inferior vena cava. From the spinal attachment point, the psoas major muscle was slowly stripped, and the L4/5 and L5/6 intervertebral disks (iliac crest flat to the L5/6 intervertebral disk or L6 vertebral body) were exposed. After identifying the annular fiber, a syringe was used to break the annulus. After successful puncture, the organ was checked to ensure that there was no bleeding and that the peritoneum was intact. Then, the animal was thoroughly treated with iodophor, and an appropriate amount of cefuroxime sodium was applied to the wound to prevent infection, and the muscle, fascia and skin were closed layer by layer.

### Groupings and processing

Thirty rats were randomly divided into a blank group (*n* = 10), a model group (*n* = 10) and a Chinese medicine group (*n* = 10). The blank group consisted of healthy rats without specific treatment. The IDD model group was subjected to annulus fibrosus puncture, and the traditional Chinese medicine group was given Yougui Wan by gavage. According to "the ratio of equivalent dose to body surface area between human and animal", the dosage of Yougui Wan was approximately 10.48 g/kg [15] after converting the standard adult body weight (70 kg) to the equivalent medium dose to the body surface area of rat. After two weeks, the rats were killed by cervical dislocation, and abdominal aorta blood and L4/5 and L5/6 intervertebral disk tissues were taken for subsequent analysis.

### Detection indicators

Blood (5 mL) was collected from the abdominal aorta and centrifuged for 15 min after standing at room temperature for 30 min. The speed was set to 3000 r/min, and the serum was stored at − 80 °C for later use. Serum levels of IL-10, MIF and TNF-*α* were determined by ELISA kits, and the process was carried out according to the kit instructions.

Proteins were extracted from the tissue samples, and then 10% SDS‒PAGE was used to separate the protein samples, which were transferred to PVDF membranes. The membranes were immersed in buffer, shaken for 1 h, and then incubated with primary antibodies at 4 °C overnight. After the membranes were washed 3 times with TBST, the secondary antibodies were added and incubated at room temperature (25–27 °C) in the dark for 50 min. The PVDF membranes were immersed in ECL color solution for 1 min, exposed, developed and fixed in a dark room. The gray value of each band was measured by Quantity One v.4.6.2 software to obtain the gray value of each protein. Relative expression level of the target protein = gray value of the target protein/gray value of GAPDH.

Apoptosis was detected by a one-step TUNEL assay. The cells in each group were treated according to the instructions of the kit, the nuclei were stained with DAPI, and then fluorescent photographs were taken with a fluorescence microscope. Positive cells and the total number of cells in each group were counted, and the apoptosis rate in each group was calculated by determining the ratio of positive cells to the total number of cells.

### Statistical methods

EXCEL was used to establish a database, sort the data and enter it. IBM SPSS 26.0 was used for statistical analysis. Measurement data are expressed as the mean ± standard deviation ($\overline{x}$ ± s). According to the homogeneity of variance and normal distribution of data, t tests and repeated measurement analysis of variance were used for comparisons. If the data did not conform to a normal distribution, the rank sum test was used. *P* < 0.05 was considered statistically significant.

## Results

### Comparison of serum inflammatory factors in each group

Compared with those in the blank group, serum levels of the inflammatory factors IL-10, MIF and TNF-*α* in the model group and the Chinese medicine group were increased (Table 1) (*P* < 0.05). Compared with those in the model group, serum levels of the inflammatory factor IL-10 in the Chinese medicine group were increased, and the levels of MIF and TNF-*α* were decreased (*P* < 0.05).

**Table 1:** Comparison of serum inflammatory factors in each group

Group	*n*	IL-10 (pg/ml)	MIF (pg/ml)	TNF-*α* (pg/ml)
Blank group	10	22.27 ± 2.84	0.30 ± 0.03	12.48 ± 3.02
Model group	10	30.88 ± 1.68^*^	1.27 ± 0.25^*^	52.59 ± 1.51^*^
TCM group	10	53.21 ± 7.48^*#^	0.63 ± 0.21^*#^	34.88 ± 3.19^*#^

Compared with the blank group, *P < 0.05, compared with the model group, ^#^*P* < 0.05

### Protein expression levels of collagen II and Notch1 in intervertebral disk tissues in each group

**Fig. 1:**
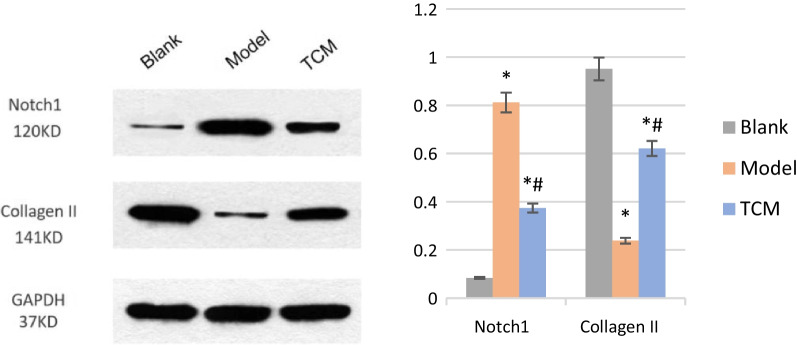
Collagen II and Notch1 protein expression levels in the intervertebral disk tissues in each group. *Note*: Compared with the blank group, ^*^P < 0.05, compared with the model group, ^#^*P* < 0.05

Compared with the blank group, the expression level of Notch1 protein in the model group and the TCM group was significantly increased, and the expression level of collagen II protein was significantly decreased. Compared with the model group, the expression level of Notch1 protein in the TCM group was significantly decreased, and the expression level of collagen II protein was significantly increased (Fig. 1). In conclusion, the expression levels of Notch1 protein in rat intervertebral disks were as follows: model group > TCM group > blank group. The protein expression of collagen II in rat intervertebral disks was as follows: blank group > TCM group > model group.

### The apoptosis rates of nucleus pulposus cells in the intervertebral disk tissues in each group

**Fig. 2:**
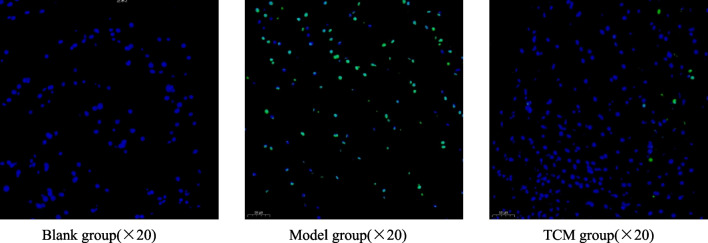
Comparison of the apoptosis rate of nucleus pulposus cells in the intervertebral disk tissues of each group. *Note*: The dots in blue are the non-apoptotic cells, and the dots in green are the apototic cells

Compared with that in the blank group, the apoptosis rate of nucleus pulposus cells in the model group and the TCM group was increased (*P* < 0.05). Compared with that in the model group, the apoptosis rate of nucleus pulposus cells in the TCM group was markedly decreased (Fig. 2, Table 2) (*P* < 0.05).

**Table 2:** Comparison of the apoptosis rate in each group

Group	*N*	Apoptosis rate
Blank group	10	9.10 ± 4.20
Model group	10	68.50 ± 11.49^*^
TCM group	10	38.10 ± 9.49^*#^

Compared with the blank group, *P < 0.05, compared with the model group, ^#^*P* < 0.05

## Discussions

This experiment explored the mechanism of Yougui Wan on degenerative intervertebral disks in rats via the Notch pathway. The ELISA results showed that Yougui Wan could reduce the levels of MIF and TNF-*α* in the serum of IDD rats and increase the levels of IL-10, indicating that Yougui Wan could significantly reduce the infiltration of inflammatory cells. The Western blot results showed that Yougui Wan could upregulate the protein expression of collagen II in intervertebral disks and reduce the protein expression of Notch1. This result suggests that Yougui Wan can alleviate the degradation of extracellular matrix, maintain normal metabolism of the nucleus pulposus, and regulate the Notch pathway. The TUNEL results showed that Yougui Wan could reduce apoptosis in nucleus pulposus cells caused by IDD.

IDD often manifests as low back pain and dysfunction. In the theory of traditional Chinese medicine, IDD belongs to the category of “arthralgia syndrome” and “low back pain.” *Suwen·stabbed Waist Pain* states that “The pulse of hengluo causes people to have low back pain, which cannot be pitched up. Pationts fear falling when leaning back. Heavy lifting or an injury to the waist could cause the disease.” The Governor Vessel runs posteriorly along the interior side of the spinal column, which is the master of yangqi. Yangqi is the main driving force for normal body function, leading to warmness, promoting blood and water, and serving as the main driving force for the operation of the body. Zhang Jingyue, a famous doctor in the Ming Dynasty, believed that “Yang is moving and dispersive, so it transforms qi, and Yin is static and stagnation, so it shapes into configuration.” A deficiency of yangqi and an excess of yinqi, cause the inability of qi to diffuse from the whole body, cause the accumulation of water, dampness, phlegm and blood stasis in the body, and then result in IDD. IDD is characterized by an accumulation in the spine and even the formation of protrusions, hypertrophy of the ligamentum flavum and hyperplasia of the articular process due to “yin shapes into configuration” [16]. Therefore, the generation, prognosis and outcome of IDD are mediated by an imbalance in yin-yang and the marker phenomenon of blood stasis. In the early stage of the disease, the deficiency of kidney yang and the decline of yangqi function account for a large proportion of the effects. Yougui Wan, which is a representative classical prescription of the warming method, takes warming kidney yang as the foundation to increase the power of Yangqi and promotes blood circulation, removing blood stasis and promoting qi to disperse the evil of tangible knots.

The Notch signaling pathway, a highly conserved signal transduction pathway regulated by the interaction between adjacent cells, is essential for bone development, plays a role in the proliferation and differentiation of endplate chondrocytes and is related to the repair of intervertebral disk cells [17–19]. In human degenerated intervertebral disks, the expression of Notch receptors and target genes was observed, and the levels of Notch signaling protein increased [20]. Compared with that in nondegenerative intervertebral disk samples, the expression level of the Notch receptor in the nucleus pulposus tissue of middle-aged degenerative intervertebral disks was increased. In addition, there is no blood supply in the intervertebral disk and a low oxygen content; thus, the capillary network in the endplate is the main source of its nutrient supply. In the early stage of IDD, the blood supply of the cartilage endplate is reduced, and the neovascular network is able to increase the nutrient supply and repair damage. The Notch pathway can regulate angiogenesis together with vascular endothelial growth factor and participate in IDD [21]. The TUNEL results showed that Yougui Wan reduced the apoptosis rate of nucleus pulposus cells in degenerative intervertebral disks. Western blot results showed that the level of Notch1 protein was significantly increased in the model group and the TCM group, and the increase in the degeneration group was more obvious, which may represent a compensatory response of the permanent cells trying to repair the degenerated intervertebral disc. Therefore, it is still necessary to pay attention to and improve the comparison of Notch1 protein content on different days in the future. To further clarify the dynamic relationship between the Notch1 protein and intervertebral disk degeneration. In addition, Yougui Wan can increase the expression of collagen II in the degenerative intervertebral disks, so as to play a protective role in the degenerative intervertebral disks.

IL-10, TNF-*α* and MIF are closely related to inflammation. IL-10 plays an immunoregulatory role in innate immunity and adaptive immunity. In innate immunity, IL-10 maintains immune homeostasis and inhibits proinflammatory responses by inducing an immunosuppressive response. In adaptive immunity, anti-inflammatory effects can be exerted by inhibiting the proinflammatory effects of CD4 + T cell subsets and promoting the stability of TR1 cells. By inhibiting the production of proinflammatory factors and chemokines, IL-10 can regulate the activity of macrophages, which is closely related to the progression of IDD and shows direct effects on intervertebral disks and neuroprotection [22, 23]. The results of this experiment showed that the serum IL-10 concentration in the model group was higher than that in the blank group, which may represent an attempt by the degenerated disk to repair itself. Yougui Wan could significantly amplify this process, further inhibit the proinflammatory effect, and delay the progression of IDD. In addition, TNF-*α* is mainly synthesized by mononuclear macrophages. When the level of TNF-*α* abnormally increases, it can accelerate ECM degradation and promote the inflammatory response [24]. MIF is a multifunctional proinflammatory factor that can antagonize the immunosuppressive effect of glucocorticoids, regulate the cell cycle and is closely related to mitophagy and cell senescence [25, 26]. The response of inflammatory cytokines can increase the expression of the Notch pathway, promoting the recovery of endogenous cells in the nucleus pulposus and compensating for apoptotic nucleus pulposus cells. However, when the level of inflammatory factors is excessively high, a cascade reaction will occur, which will further aggravate symptoms and lead to neuronal damage or even death [27]. The results of this experiment showed that the serum TNF-*α* and MIF concentrations of the TCM group were still higher than those of the blank group, and were significantly lower than those of the model group. Therefore, Yougui Wan can reduce the inflammatory cytokine-mediated destruction of intervertebral disks by inhibiting inflammation.

In summary, Yougui Wan can reduce the inflammatory response of IDD rats, maintain the balance of extracellular matrix metabolism, reduce apoptosis in nucleus pulposus cells, and delay IDD, and the mechanism may be related to the Notch pathway.

## Data Availability

The datasets generated or analyzed during this study are available from the corresponding author on reasonable request.
